# A life course perspective on working beyond retirement—results from a longitudinal study in the Netherlands

**DOI:** 10.1186/s12889-016-3174-y

**Published:** 2016-06-10

**Authors:** Astrid de Wind, Suzan van der Pas, Birgitte M. Blatter, Allard J. van der Beek

**Affiliations:** Department of Public and Occupational Health, the EMGO+ Institute for Health and Care Research, VU University Medical Center, Amsterdam, The Netherlands; Netherlands Organisation for Applied Scientific Research TNO, Leiden, The Netherlands; Body@Work, Research Center on Physical Activity, Work and Health, TNO-VU/VUmc, Amsterdam, The Netherlands; Department of Epidemiology and Biostatistics, the EMGO+ Institute for Health and Care Research, VU University Medical Center, Amsterdam, The Netherlands

**Keywords:** Ageing, Bridge employment, Employment participation, Retirement, Predictor

## Abstract

**Background:**

There is a societal need that workers prolong their working lives. By adopting a life course perspective, this study aimed to investigate the influence of work motives and motivation, health, job characteristics, skills, and financial and social situation on working beyond retirement, and differences between ‘on time’ and ‘off time’ retirees (retirement age 65 and <65 years, respectively).

**Methods:**

Retirees aged 57 to 67 years (*N =* 1,054) who participated in the Dutch Study on Transitions in Employment, Ability and Motivation were included in this study. Participants filled out a questionnaire in 2010, 2011, 2012, and 2013. Predictors of working beyond retirement were identified using logistic regression analyses, and stratified analyses were performed to investigate differences between ‘off time’ and ‘on time’ retirees.

**Results:**

High work engagement (OR = 1.3), good physical health (OR = 1.8), poor financial situation (OR = 2.4), and voluntary work (OR = 1.5) predicted working beyond retirement. For ‘off time’ retirees, no financial possibility to retire early (OR = 1.8) and not having a partner (OR = 1.9) predicted working beyond retirement. ‘On time’ retirees reporting more support at work (OR = 0.7) and without the financial possibility to retire early (OR = 0.5), worked beyond retirement less often.

**Conclusions:**

The results indicated that especially the motivation to work, physical health and the financial situation were the most relevant aspects with regard to working beyond retirement, which supports the idea that the principle of ‘human agency’ of the life course perspective is useful to understand factors that impact working beyond retirement. Most aspects of the life course principles of ‘linked lives’ and ‘timing’ seemed to be less relevant.

## Background

To encounter the pressure of population ageing on the social security system and the expected shortage of workers in the next decades in many developed countries [1], there is a societal need for workers to prolong their working life. Many European governments responded to this need by increasing the statutory retirement age and discouraging early exit from the workforce. Working beyond retirement may also provide in the need for prolonged working lives. Although previous research has identified a variety of factors that influence early exit from the labor market, still little is known about the factors that impact working beyond retirement.

Retirement does not necessarily mean a final farewell to the labor market. In the Netherlands, a growing number of retirees engage in work activities between the end of their career employment and complete labor force withdrawal, which is often referred to as bridge employment. The percentage of people in the age group 65 to 70 years that works for at least 12 h per week, increased from 3.4 % in 2001 to 8.6 % in 2014 [2]. In comparison, these numbers are much higher in the United States where 30.8 % of the age group 65 to 69 worked at least 35 h per week, in 2010 [3].

Although previous research has shown that health, job characteristics, skills and knowledge, and financial and social factors may push or pull workers from work to (non-disability) early retirement [4–10], still little is known about the factors that impact working beyond retirement. It is unclear whether this is just predicted by a beneficial status of factors that influence early retirement, or whether also additional factors play a role. Second, it is unclear whether different factors predict working beyond retirement among retirees who retire ‘on time’ compared to retirees who retire early. Third, occupational epidemiologists often focus on health, work ability and physical job demands in relation to work force participation, whereas psychologists focus on motivational processes and psychosocial job demands, and economists focus on financial factors. To understand the complexity of working beyond retirement a broader perspective is needed.

### Theoretical background

The life course perspective provides the opportunity to get a better understanding of the different factors that influence working beyond retirement. According to this perspective, it is expected that transitions, i.e. changes related to entering or exiting roles, such as the transition from work to retirement and vice versa, are embedded within multiple interdependent trajectories, i.e. within the life spheres health, work, family and leisure [11]. Working beyond retirement cannot be fully understood in isolation of a person’s health, work and motivation to work, skills and knowledge, and financial and social situation (i.e. family and other forms of social participation). In the present study we will use the life course perspective as a framework to understand determinants of working beyond retirement by focusing on the life course principles ‘human agency’, ‘linked lives’, and ‘timing’ [11].

‘*Human agency’* implies that individuals actively create their own lives and choices, but that this is done within a set of opportunities and constraints that come with one’s history or that exist in one’s environment. Previous research showed that work motivation and motives are important predictors of early retirement intention and working beyond retirement, respectively [12, 13]. A study showed that the most important reason to work beyond retirement, is that someone enjoys working [13]. Health problems can be considered as a constraint to continue working, since it predicts early retirement [10, 14]. Job characteristics, such as physical work demands and high work pressure, may also hinder continued working [8, 15]. On the other hand, psychosocial job characteristics, such as appreciation at work, could facilitate continued working [5]. Financial factors can also be considered as a constraint or an opportunity to stop working. Previous research showed that the financial possibility to retire early strongly contributed to early retirement [5].

The life course perspective also emphasizes the social embeddedness of transitions, by pointing to the principle of *‘linked lives’*. This principle refers to the interdependence of lives and it states that the lives of individuals are bound to the lives of others [11]. Transitions from work to early retirement are shaped to a large extent by social relationships, i.e. within the family and at work. Previous research showed that having a partner increased the likelihood of early retirement [8]. Also support of the partner to continue working or to retire early predicted retirement at older age and early retirement [5, 16].

Another principle within the life course perspective is ‘*timing’*, which refers to the age at which an experience occurs and how it is experienced [11]. Transitions can occur ‘on time’ or ‘off time’. Transitions that occur off time ensure that persons do not have the chance to go through anticipatory socialization, and that they lack peers to provide social support and to share experiences regarding the transition [11]. Therefore, 'off time' transitions might be experienced as involuntary. In recent decades, there has been a strong ‘early exit culture’ in the Netherlands [17]. However, several pension system reforms have been implemented, such as the increase of the statutory retirement age from 65 years in 2012 to 67 in 2021 [18]. In addition, there is an extensive public debate on the need to prolong working life, and hence, the social norm may have become more favorable with respect to prolonged careers. Retiring at the statutory retirement age can be considered as ‘on time’ retirement, and retiring before the statutory retirement age can be considered as ‘off time’ retirement. Although early retirement is often considered as one of the more ‘voluntary’ pathways of early exit from the workforce, especially as compared to disability pension and unemployment, a recent qualitative study showed that early retirement is not always experienced in this way [19]. In this study employees with poor health sometimes retired early because they felt pushed out by their employer, although they themselves did not experience a reduced ability to work. In addition, such involuntary transitions from work to early retirement may have negative financial consequences, in situations where there was actually a financial need to continue working until the statutory retirement age.

### Research questions and hypotheses

In the present study we investigate which factors influence working beyond retirement from a life course perspective. We aim to gain insight in how the timing of retirement could define subgroups of retirees regarding influential factors. Our first research question is: What is the influence of individual characteristics, work motives and motivation, health, job characteristics, skills and knowledge, and the financial and social situation on working beyond retirement? Our second research question is: Are there differences in the factors which influence working beyond retirement between those retirees who reached the statutory retirement age of 65 years, and thus retired ‘on time’, and those who did not yet reach the statutory retirement age, and thus retired ‘off time’ (early)? In line with the principle ‘timing’ of the life course perspective, we expect that ‘on time’ retirees are intrinsically motivated to work beyond retirement, e.g. because they enjoy working, find their work meaningful or interesting, or think it offers opportunities for learning and development, whereas ‘off time’ retirees are extrinsically motivated to work beyond retirement, e.g. for financial reasons or because others expect them to do so.

## Methods

### Design and study population

The current study is part of the Study on Transitions in Employment, Ability and Motivation (STREAM) [20]. STREAM is a Dutch longitudinal study among 15,118 persons including employees (*N =* 12,055), self-employed persons (*N =* 1,029), and persons without paid employment (*N =* 2,034) aged 45 to 64 years [20]. Persons participated in the GfK Intomart online panel and yearly filled out an online questionnaire in October/November 2010 (T1), 2011 (T2), 2012 (T3), and 2013 (T4). Participants come into the internet panel in various ways: via national representative research carried out by GfK Intomart (33 %), via contacts of persons already included in the panel (23 %), via newsletters (26 %), via banners (2 %), or because they applied for the internet panel themselves (16 %). They were paid to complete a questionnaire, i.e. for every completed questionnaire, the savings balance of the participant was increased by about €3.00. Persons who participated at baseline received all follow-up questionnaires, except for those who stated they did not want to participate in the GfK Intomart panel and on-going studies anymore. Approaching participants for the follow-up questionnaires took place in the same way as for the baseline questionnaire. The study population of STREAM, including the way of approaching participants, was previously extensively described elsewhere [20]. In the present study, we used data of all waves of STREAM.

**Fig. 1:**
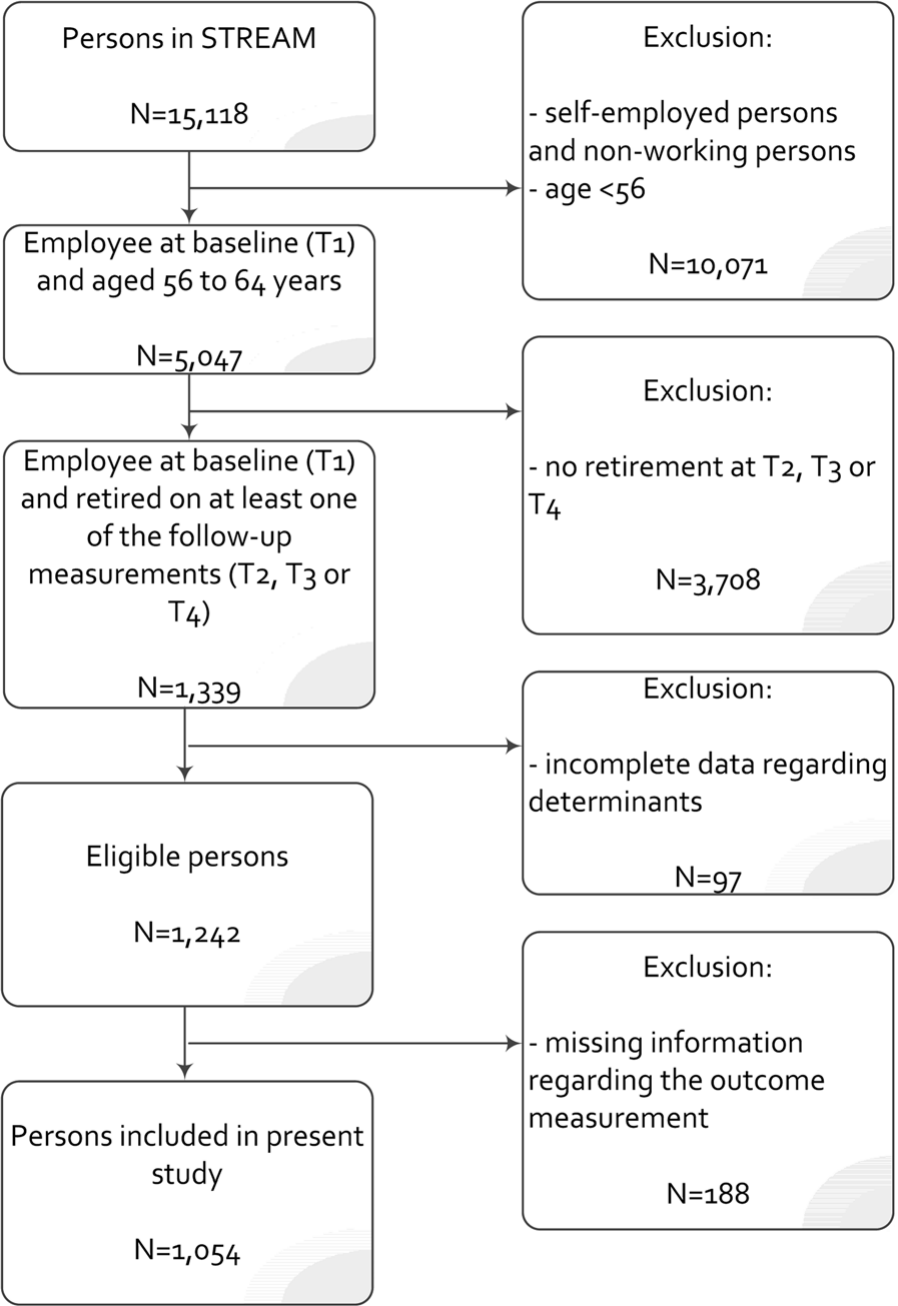
Flow of the study population

The study population of the present study consisted of persons who were employee at baseline (T1), who retired (‘on time’ or ‘off time’) at one of the follow-up measurements (T2/T3/T4) and who were aged 56 to 64 years at baseline (T1). We chose 56 years as a lower age limit, since the proportion of employees that had retired (early) after one, two or three years of follow-up strongly increased from this age onwards. 64 years was the upper age limit, because this is the maximum age of persons included in STREAM at baseline. Persons who retired (early) at baseline were excluded, since we were interested in job characteristics as one of the domains of determinants in the present study. Also persons who lacked information on one of the determinants or the outcome variable were excluded from the present study. In total, 1,054 persons were included (Fig. 1). To determine whether loss to follow-up was selective, we compared baseline characteristics of non-responders and responders using independent *t*-test. At baseline persons loss to follow-up (i) were more often women (47 % versus 38 %), (ii) had more often low or medium educational level (low in 35 % versus 29 %, medium in 36 % versus 33 %, and high in 28 % versus 37 %), and (iii) reported a slightly lower focus on development of knowledge and skills (3.7 versus 3.8).

### Measurements

All independent variables were derived from the baseline questionnaire and the outcome variable, i.e. working beyond retirement, was derived from the follow-up questionnaires.

#### Working beyond retirement

The outcome measure of the present study was working beyond retirement on one of the follow-up measurements. Working beyond retirement referred to working as an employee or self-employed person, while also receiving some form of retirement pension (old age pension or pension via an early retirement scheme). Those who worked next to receiving a pension were compared with those who received a pension and did not work anymore.

Information on work and retirement was derived from one question asking persons to indicate their employment status with, among others, the following answering options: a paid job or multiple paid jobs as an employee, self-employed, early retirement and retirement. The Dutch pension system consists of three pillars: the state old-age pension, supplementary pension schemes by virtue of the employer or sector (about 90 % of all employees), and private savings. The statutory retirement age at which persons receive their state old-age pension was raised from 65 years in 2012 to 67 in 2021. In this study retirement referred to employees who were aged 65 years or older and that they retired. Early retirement referred to those who reported that they retired early or those under the age of 65 years, whereas they reported that they retired.

Persons with and without an interruption of not working immediately after retirement fall within this definition. We do not know the percentages of persons who continued working without an interruption and who started working after an interruption of not working, but in a subsample of persons who worked beyond retirement and who participated in all four measurements, the percentages were 61 %, and 39 % respectively.

#### Individual characteristics

Educational level was measured using a question on the highest level of education completed with a diploma, and categorized into low (primary school, lower and intermediate secondary education, or lower vocational training), intermediate (higher secondary education, or intermediate vocational training) or high (higher vocational education or university).

Mastery was measured using the Pearlin Mastery Scale, which reflects the degree to which persons feel they are in control of matters that affect their lives [21]. This scale consists of seven items with a 5-point answering scale ranging from 'totally disagree' to 'totally agree' (Cronbach’s alpha 0.84). A higher score reflects a higher degree of mastery. Mastery was analyzed as a continuous scale.

#### Work motives and motivation

The following work motives were measured: working because someone likes to work, working because someone finds work meaningful, working for financial reasons, and working because others expect them to do so. Working because someone likes to work was measured using a scale consisting of three items (Cronbach’s alpha 0.72). Working because someone finds work meaningful was measured using a scale consisting of two items (Cronbach’s alpha 0.81). Working for financial reasons was measured using one item: 'I work to earn money'. Working because others expect them to do so was measured with one item: 'I work because people in my environment think it is important'. All items on work motives could be answered on a 5-point answering scale ranging from 'totally disagree' to 'totally agree'. Due to the skewed distribution of the two scales and the two separate items on work motives, the variables were dichotomized.

Work engagement was measured using the dimensions vigor (three items) and dedication (three items) of the Utrecht Work Engagement Scale (UWES) [22]. Vigor refers to having a lot of energy at work and mental resilience, feeling strong and fit, and not getting tired from work very fast. Dedication refers to enthusiasm, inspiration, proud, and job satisfaction. The dimensions vigor and dedication were combined to one scale for work engagement (Cronbach’s alpha = 0.93). Items could be answered on a 7-point scale (‘never’ to ‘always’) and a higher score reflects a higher work engagement. In the analyses we considered work engagement as a continuous variable.

#### Health

Perceived physical health and perceived mental health was measured using the physical component summary scale (PCS) and the mental component summary scale (MCS) of the Short Form-12 Health Survey respectively [23]. The scales range from 0-100 (0 = worst and 100 = best possible health status). An example of a PCS item: 'Does your health now limit you in climbing several flights of stairs?'. An example of a MCS item is: 'Have you felt downhearted and blue?'. Due to the skewed distribution of both scales, the interquartile range (25th–75th percentile) was used to distinguish between poor, moderate, and good health.

#### Job characteristics

Physical demands were measured using a scale consisting of six items on regular use of force, the use of vibrating tools, awkward postures, prolonged standing, and prolonged squatting based on the Netherlands Working Conditions Survey 2009 [24] and the Dutch Musculoskeletal Questionnaire (Cronbach’s alpha 0.86) [25]. A 5-point answer scale was used ranging from 'always' to '(almost) never'. Due to the skewed distribution, the interquartile range was used to distinguish between high, moderate, and low physical demands.

Job demands and job autonomy were measured using four and five items, respectively, all derived from the Job Content Questionnaire (Cronbach’s alpha 0.87 and 0.78, respectively) [26, 27]. A 5-point scale was used ranging from 'always' to '(almost) never'. An example of a job demands item is 'Do you have to work very fast?'. An example of an autonomy item is 'Are you able to decide for yourself how to do your work?'. Higher scores reflect higher job demands and higher job autonomy. Job demands and job autonomy were analyzed as continuous scales.

Furthermore, employees indicated on a 4-point scale whether the following aspects are present at work: appreciation, interesting work, and opportunities for learning and development [28]. The answers 'not present at all' and 'somewhat present' were classified into 'not present' and 'rather present' and 'highly present' were classified into 'present'.

In addition, social support of colleagues and supervisor was measured using a 4-item scale derived from the Copenhagen Psychosocial Questionnaire (COPSOQ) (Cronbach’s alpha 0.81) [29]. Employees indicated how often their colleagues or their supervisor helped or supported them and how often they were willing to listen to their work-related problems. A 5-point answer scale was used ranging from 'always' to 'almost never'. Lower scores reflect higher social support from colleagues and supervisor. Social support of colleagues and supervisor was analyzed as a continuous scale.

#### Skills and knowledge

Developmental proactivity was measured using a 4-item scale derived from Van Veldhoven and Dorenbosch [30]. This scale reflects the extent to which persons (i) actively search for activities in their job that allow them to expand knowledge and skills and (ii) adapt their knowledge and skills to (future) changes in their jobs. Items could be answered on a 5-point scale ('totally disagree' to 'totally agree' and Cronbach’s alpha was 0.81). A higher score means a higher focus on development of skills and knowledge. Developmental proactivity was analyzed as a continuous scale.

#### Financial situation

In the domain of financial situation, the financial situation of the household was measured using the following item: ‘What is the financial situation of your household now?’. Answering categories were 'very short of money', 'somewhat short of money', 'just adequate', 'some money left' and 'a lot of money left'. The categories were classified into 'money left', 'just adequate', or 'short of money'. Furthermore, the financial possibility to stop working before age 65 was measured. Employees were asked: 'Could you financially afford to stop working before the official retirement age?' This question could be answered with 'yes', 'no', or 'don’t know'.

#### Social situation

Participants provided information on their household composition. In the analyses we distinguished between persons with and without a partner. Also, employment status of a partner was assessed. In the analyses we distinguished between non-working and working. In addition, participation in informal care and voluntary work were assessed.

### Analysis

Regarding the first research question predictors of working beyond retirement (no/yes) were studied by logistic regression analyses. Odds ratios (OR) and 95 % confidence intervals (95 % CI) were calculated to express the probability of working beyond retirement. In the first step of the analyses, univariate associations between individual characteristics, work motives and motivation, health, job characteristics, skills and knowledge, and financial and social situation at T1 with working beyond retirement on one of the follow-up measurements (T2/T3/T4) were established. Second, multivariate analyses were performed for all variables in the univariate analyses with *p <* 0.20, using stepwise backward elimination. Variables with *p <* 0.05 were retained in the final multivariate model. Age and gender were included in all analyses by default.

To answer the second research question about differences in determinants of working beyond retirement between persons who retired ‘off time’ and persons who retired ‘on time’ we performed stratified analyses for these groups. We followed the same procedure as for the whole group. Groups were defined on the basis of timing of their retirement: ‘off time’ retirement for persons who retired before the age of 65 or ‘on time’ retirement for persons who were 65 when they retired. All statistical analyses were carried out using SPSS Statistics 22.

### Ethical issues

The Medical Ethical Committee of the VU University Medical Center Amsterdam declared that the Medical Research Involving Human Subjects Act (abbreviation in Dutch: WMO) did not apply to STREAM. The Medical Ethical Committee had no objection to the execution of this study. In the information for participants that accompanied the online questionnaire, it was emphasized that the privacy of participants was guaranteed, that all answers to the questions were treated confidentially, and that all data were stored in secured computer systems.

### Availability of data

Data that were used for the present study are available on request (infostream@tno.nl).

## Results

Table 1 shows the characteristics of the study population (*N =* 1,054). In total, 25 % of the employees at T1 that retired at T2, T3 or T4, worked beyond retirement (*N =* 264). 137 persons worked beyond ‘off time’ retirement and 127 persons worked beyond ‘on time’ retirement.

**Table 1:** Characteristics of the study population (*N =* 1,054)

Characteristics		Frequency (%)	Mean	IQR*
Individual characteristics				
Age	56–64 years	-	61.3	60.0–63.0
Gender	Male	61.6	-	-
Educational level	Low	29.1	-	-
	Intermediate	33.5	-	-
	High	37.4	-	-
Mastery	1–5	-	3.8	3.4–4.1
Work motives and motivation				
Working because someone likes to work	Yes	28.9	-	-
Working because someone finds work meaningful	Yes	26.9	-	-
Working for financial reasons	Yes	41.4	-	-
Working because others expect them to do so	Yes	23.9	-	-
Work engagement	1–7	-	4.4	3.7–5.3
Health				
Physical health	Poor	25.2	-	-
	Moderate	46.5	-	-
	Good	28.3	-	-
Mental health	Poor	25.3	-	-
	Moderate	49.8	-	-
	Good	24.9	-	-
Job characteristics				
Physical demands	High	22.1	-	-
	Medium	38.3	-	-
	Low	39.6	-	-
Job demands	1–5	-	3.0	2.5–3.5
Autonomy	1–5	-	3.9	3.4–4.4
Social support	1–5	-	3.5	3.0–4.0
Appreciation	Present	56.5	-	-
Interesting work	Present	22.6	-	-
Opportunities for learning and development	Present	7.2	-	-
Skills and knowledge				
Developmental proactivity	1-5	-	3.8	3.5–4.0
Financial situation				
Financial situation of the household	Money left	66.6	-	-
	Just adequate	22.7	-	-
	Short of money	10.7	-	-
Financial possibility to stop working before age 65	Yes	62.9	-	-
	No	29.2	-	-
	Don’t know	7.9	-	-
Social situation				
Partner	No	22.5	-	-
Employment status partner	Not working	39.0	-	-
	Working	38.5		
	No partner	22.5		
Informal care	Yes	19.9	-	-
Voluntary work	Yes	41.0	-	-
Outcome				
Working beyond retirement	Yes	25.0	-	-

*Interquartile range (25^th^-75^th^ percentile)

**Table 2:** Longitudinal associations between determinants and working beyond retirement in logistic regression analyses (*N =* 1,054)

Characteristics		Univariate		Multivariate	
		OR	95 % CI	OR	95 % CI
Individual characteristics					
Age	56–64 years	1.1**	1.0–1.2	1.1**	1.0–1.2
Gender	Male	1.3*	0.9–1.7	1.4**	1.0–1.9
Educational level	Low	1.0			
	Intermediate	1.1	0.8–1.6		
	High	1.2	0.9–1.7		
Mastery	1–5	1.2*	1.0–1.6		
Work motives and motivation					
Working because someone likes to work	Yes	1.4**	1.1–2.0		
Working because someone finds work meaningful	Yes	1.1	0.8–1.5		
Working for financial reasons	Yes	1.0	0.7–1.3		
Working because others expect them to do so	Yes	1.1	0.8–1.5		
Work engagement	1–7	1.4**	1.2–1.5	1.3**	1.1–1.5
Health					
Physical health	Poor	1.0		1.0	
	Moderate	1.2	0.8–1.7	1.1	0.7–1.6
	Good	2.1**	1.4–3.1	1.8**	1.2–2.8
Mental health	Poor	1.0			
	Moderate	1.4*	1.0–2.0		
	Good	1.5*	1.0–2.2		
Job characteristics					
Physical demands	High	1.0			
	Medium	1.4*	0.9–2.0		
	Low	1.3*	0.9–1.9		
Job demands	1-5	1.0	0.8–1.2		
Autonomy	1-5	1.0	0.9–1.3		
Social support	1-5	0.9	0.8–1.1		
Appreciation	Present	1.4**	1.1–1.9		
Interesting work	Present	1.7**	1.3–2.4		
Opportunities for learning and development	Present	1.3	0.8–2.2		
Skills and knowledge					
Developmental proactivity	1-5	1.5**	1.2–1.9		
Financial situation					
Financial situation of the household	Money left	1.0			
	Just adequate	0.8	0.6–1.2	1.0	0.7–1.4
	Short of money	2.0**	1.3–3.0	2.4**	1.5–3.7
Financial possibility to stop working before age 65	Yes	1.0			
	No	1.1	0.8–1.5		
	Don’t know	0.9	0.5–1.6		
Social situation					
Partner	No	1.1	0.8–1.6		
Employment status partner	Not working	1.0			
	Working	1.1	0.8–1.6		
	No partner	1.2	0.8–1.8		
Informal care	Yes	0.8	0.5–1.1		
Voluntary work	Yes	1.4**	1.1–1.9	1.5**	1.1–2.0

*p value < 0.20, **p value < 0.05

### Determinants of working beyond retirement

In the univariate logistic regression analyses, older persons, those who work because they like to work, and those with higher degrees of work engagement, appreciation at work and interesting work, those with good physical health, those with a high focus on development of skills and knowledge (i.e. developmental proactivity), those who participate in voluntary work and those who have a poor financial situation, worked beyond retirement more often (Table 2). In the multivariate analyses, older age (OR = 1.1), being male (OR = 1.1), high work engagement (OR = 1.3), good physical health (OR = 1.8), participating in voluntary work (OR = 1.5) and a poor financial situation (OR = 2.4), predicted working beyond retirement.

**Table 3:** Longitudinal associations between determinants and working beyond ‘off time’ (*N =* 638) and ‘on time’ retirement (*N =* 416) in logistic regression analyses

Characteristics		‘Off time’ retirement				‘On time’ retirement			
		Univariate		Multivariate		Univariate		Multivariate	
		OR	95 % CI	OR	95 % CI	OR	95 % CI	OR	95 % CI
Individual characteristics									
Age	56-64 years	1.0	0.9–1.1	1.0	0.8–1.1	1.3**	1.1–1.7	1.3**	1.0–1.6
Gender	Male	2.0**	1.3–3.0	2.1**	1.3–3.3	0.9	0.6–1.4	0.9	0.6–1.4
Educational level	Low	1.0				1.0			
	Intermediate	1.4	0.8–2.3			0.9	0.6–1.6		
	High	1.4*	0.9–2.3			1.2	0.7–2.0		
Mastery	1-5	1.1	0.8–1.5			1.5**	1.0–2.1		
Work motives and motivation									
Working because someone likes to work	Yes	1.6*	1.0–2.4			1.2	0.8–1.8		
Working because someone finds work meaningful	Yes	1.0	0.6–1.6			1.2	0.7–1.8		
Working for financial reasons	Yes	1.1	0.8–1.6			0.8	0.5–1.2		
Working because others expect them to do so	Yes	1.5*	1.0–2.2			0.8	0.5–1.3		
Work engagement	1-7	1.2**	1.0–1.4	1.3**	1.1–1.5	1.5**	1.2–1.8	1.5**	1.2–1.9
Health									
Physical health	Poor	1.0		1.0		1.0			
	Moderate	1.2	0.7–2.0	1.2	0.7–2.0	1.1	0.6–1.9		
	Good	2.0**	1.2–3.4	2.1**	1.2–3.8	2.2**	1.2–3.8		
Mental health	Poor	1.0				1.0			
	Moderate	1.2	0.8–1.9			1.8*	1.0–3.2		
	Good	1.0	0.6–1.7			2.3**	1.2–4.2		
Job characteristics									
Physical demands	High	1.0				1.0			
	Medium	1.4*	0.9–2.4			1.2	0.6–2.1		
	Low	1.4	0.8–2.3			1.0	0.6–1.9		
Job demands	1-5	1.2	0.9–1.5			0.9	0.7–1.1		
Autonomy	1-5	1.3*	1.0–1.7			0.9	0.7–1.1		
Appreciation	Present	1.3*	0.9–2.0			1.5*	1.0–2.3		
Interesting work	Present	1.6**	1.0–2.5			1.8**	1.1–2.8		
Opportunities for learning and development	Present	1.9*	1.0–3.6			0.8	0.4–1.9		
Social support	1-5	1.0	0.8–1.3			0.8*	0.6–1.0	0.7**	0.6–0.9
Skills and knowledge									
Developmental proactivity	1-5	1.4**	1.0–2.0			1.5**	1.1–2.1		
Financial situation									
Financial situation of the household	Money left	1.0				1.0		1.0	
	Just adequate	0.8	0.5–1.3	0.9	0.5–1.5	0.8	0.5–1.4	1.0	0.5–1.7
	Short of money	2.2**	1.2–3.9	2.0**	1.0–4.0	1.7*	0.9–3.0	2.2**	1.1–4.4
Financial possibility to stop working before age 65	Yes	1.0		1.0		1.0		1.0	
	No	1.7**	1.1–2.8	1.8**	1.1–3.2	0.6**	0.4–0.9	0.5**	0.3–0.8
	Don’t know	1.2	0.6–2.4	1.5	0.8–3.1	0.5*	0.2–1.3	0.4*	0.1–1.1
Social situation									
Partner	No	1.3	0.8–2.1			0.8	0.5–1.3		
Employment status partner	Not working	1.0		1.0		1.0			
	Working	1.3*	0.9–2.1	1.3	0.8–2.1	1.0	0.6–1.7		
	No partner	1.6*	0.9–2.7	1.9**	1.1–3.4	0.9	0.5–1.4		
Informal care	Yes	0.8	0.5–1.2			0.9	0.5–1.5		
Voluntary work	Yes	1.6**	1.1–2.4	1.5**	1.0–2.2	1.3*	0.9–2.1		

*p value < 0.20, **p value < 0.05

### Determinants of working beyond ‘off time’ and ‘on time’ retirement

In the multivariate analyses within the group of persons that retired ‘off time’, being male (OR = 2.1), high work engagement (OR = 1.3), good physical health (OR = 2.1), participating in voluntary work (OR = 1.5) and a poor financial situation (OR = 2.0) predicted working beyond retirement (Table 3). The ORs were comparable to the ORs of the total group of retirees. In addition, not having a partner (in comparison with a non-working partner) (OR = 1.9) and not having the financial possibility to retire before the age of 65 (OR = 1.8) predicted working beyond retirement.

In the multivariate analyses within the group of persons that retired ‘on time’, older age (OR = 1.3), high work engagement (OR = 1.5) and a poor financial situation (OR = 2.2) predicted working beyond retirement (Table 3). The ORs were comparable to those of the total group of retirees. In addition, persons who experienced more social support from colleagues and supervisor (OR = 0.7) and persons who did not have the financial possibility (OR = 0.5) or did not know whether they had the financial possibility (OR = 0.5) to retire before the age of 65 worked beyond retirement less often.

## Discussion and Conclusion

This study aimed to gain insight in the influence of individual characteristics, work motives and motivation, health, job characteristics, skills and knowledge, and the financial and social situation on working beyond retirement and to investigate whether there are differences between ‘on time’ and ‘off time’ retirees.

Regarding the first research question, our study showed that work motivation, health, and the financial situation influence working beyond retirement. This is in line with the principle ‘human agency’ of the life course perspective and with previous studies on predictors of early retirement [5, 6, 9, 10, 12]. The results showed that persons who were highly engaged in their work were more likely to work beyond retirement, which confirms findings from a previous study that showed that intrinsic motivation was related to the willingness to continue working [31], and a study that showed that a lower motivation to work was associated with intention to retire early [12]. Previous research showed that self-perceived poor health predicts early retirement, but that specifically mental health problems are not related to early retirement [10]. Indeed, the opposite seems true with regard to working beyond retirement; good physical health predicts working beyond retirement and good mental health does not. Hence, even though our study population probably is a selection of the more healthy employees, since the unhealthy ones partly have left the workforce through disability benefits in previous years (‘healthy worker effect’ [32]), physical health predicts working beyond retirement. Contrary to what we expected, work motives, job characteristics, and skills and knowledge did not predict working beyond retirement multivariately, whereas the enjoyment motive, appreciation at work, interesting work and developmental proactivity predicted working beyond retirement in the univariate analyses. This may be explained by work engagement, which was moderately interrelated with these variables (Pearson’s correlations 0.36, 0.30, 0.34, and 0.40, respectively).

In line with the principle ‘linked lives’ of the life course perspective, we expected that having a partner, and employment status of the partner are related to working beyond retirement. However, our study found no effect. This contradicts previous research that showed that having a partner increased the likelihood of early retirement [8], and that the attitude and support of the partner about continuing to work or early retirement predicted retirement at older age and early retirement, respectively [5, 16]. However, persons who participated in voluntary work were more likely to work beyond retirement, which is an indication that working beyond retirement is embedded in someone’s social situation to a certain degree, and that it cannot be seen in isolation from other social relationships.

Regarding the second research question, our study showed that determinants of working beyond ‘off time’ retirement and working beyond ‘on time’ retirement were largely comparable. However, two remarkable differences should be mentioned; in the group of ‘off time’ retirees, not having the financial possibility to retire early predicted working beyond retirement, whereas in the group of ‘on time’ retirees persons who did not have the financial possibility to retire early were less likely to work beyond retirement. This partially supports our hypothesis that ‘off time’ retirees are extrinsically motivated to work beyond retirement to a larger extent than ‘on time’ retirees. This might indicate that the group of ‘off time’ retirees partly consisted of persons who experienced their transition from work to early retirement as involuntary, for example because they felt pushed out by their employer, whereas they actually wanted to continue working or could not financially afford to retire early, and therefore started to work beyond retirement. Unfortunately we did not have information on voluntariness of the transition. Future research might investigate whether the retirement context, i.e. the reason for retirement and voluntariness of retirement could be important characteristics to define subgroups of retirees with regard to working beyond retirement.

Persons who were highly engaged in their work, were more likely to work beyond retirement, both in the group of ‘off time’ retirees and the group of ‘on time’ retirees. This is contrary to our hypothesis that ‘on time’ retirees would be intrinsically motivated to a larger extent than ‘off time’ retirees. Although timing of retirement could partially define subgroups of retirees, the pattern regarding intrinsic and extrinsic motivation is not as clear as we would have expected based on the timing principle of the life course perspective. This may indicate that there are also other characteristics that could define subgroups of retirees, such as educational level. It may be that within our study population, educational level explains why we found that both intrinsic factors and extrinsic factors predicted working beyond retirement. We tested this possibility by post-hoc analyses stratified by educational level (data not shown). The results indicated that within persons with a higher educational level, having the financial possibility to retire early predicted working beyond retirement, whereas within persons with a lower educational level having the financial possibility was not related to working beyond retirement. However, the role of work engagement was comparable for persons with a low educational level and those with a high educational level. Hence, also educational level could only partially explain which factors predicted working beyond retirement. This may imply that either there are no clear subgroups of retirees regarding working beyond retirement, or that other characteristics, which were not incorporated in the present study, play a role. Although our study involved a broad range of variables in a diversity of domains, we did not consider different ‘types’ of retirement. We were not able to distinguish between retirement schemes, e.g. an early retirement scheme via employer or sector, retirement because someone reached a job-specific retirement age, part-time, or full-time retirement. Especially the degree to which working beyond retirement is financially attractive might differ between different retirement schemes. Future research might further investigate whether education and ‘type’ of retirement are relevant characteristics to define subgroups of retirees.

Strengths of the present study are the longitudinal character of the large dataset, and the fact that variables frequently studied in different areas of expertise, i.e. work motives and motivation, health, job characteristics, skills and knowledge, and the financial and social situation, were all included in this study. Moreover, we had low drop-out in the present study; 79 % of the participants of interest at T1 also participated at T2, T3, and T4, which can be considered as a high response in longitudinal research. However, this study also has limitations. First, all data relied on self-reports. A drawback of measuring employment status relying on self-report is that it is dependent on the interpretation of the participant and it is thus not defined by, for example, the main source of income as registered by a tax institution or the government. A second limitation is the relatively short follow-up period of three years, which concerns only a small part of the whole life course. From a life course perspective one might expect that also experiences earlier in life impact decisions regarding work and retirement. This was supported by a study of Damman et al. that showed that mid-life experiences, i.e. educational investments, job changes, late transitions into parenthood, and late divorces, are associated with weaker retirement intentions, whereas mid-life health problems are associated with stronger retirement intentions [4]. In addition, this study showed that later labour market entry and late transitions into parenthood were associated with a lower likelihood of early retirement, and that part-time work before the age of 50 years resulted in a higher likelihood of early retirement. Third, selection bias may have occurred as a result of selective entry in this internet panel. Certain groups of persons, i.e. without internet access, illiterate persons and persons who do not master the Dutch language, may be underexposed in an internet panel. The findings of the present study may not be generalizable to these groups. Selection bias may also have occurred as a result of selective non-response at follow-up. However, the response of the study was high, i.e. 79 %, and differences between respondents and non-respondents on baseline characteristics were small, and considered as not relevant.

As previously described, future research is needed to investigate the role of the retirement context, i.e. the reason for retirement and voluntariness of retirement, as well as differences in educational level and ‘type’ of retirement with regard to working beyond retirement. Besides, we used the statutory retirement age of 65 years to distinguish between ‘on time’ and ‘off time’ retirement. Since not only institutional but also cultural and individual norms determine the ‘right’ time of a transition, future research is needed to determine where the line should be drawn. Furthermore, future research should explore further possibilities of using the life course perspective to understand factors that influence working beyond retirement. There is still little understanding on *how* different determinants influence working beyond retirement and *why* persons work beyond retirement. Especially the group of persons who works beyond *early* retirement is of interest, since an alternative career path for these persons may have been to retire at later age. Finally, it is unclear whether working beyond retirement mostly occurs in an employment contract with same employers and occupations or different employers and occupations, and whether it can be seen as a sustainable way of prolonging working life. We recommend future research to give more insight in these characteristics of working beyond retirement.

In conclusion, our results indicated that especially the motivation to work, physical health, the financial situation of the household, and participation in voluntary work played an important role in working beyond retirement. This supports the idea that the human agency principle of the life course perspective is useful to understand factors that impact working beyond retirement. Since social situation did not play a role, except for voluntary work, less evidence was found for the linked lives principle. Also timing seemed to be less relevant, since hardly any differences between ‘off time’ and ‘on time’ retirees were found.

## References

[1] European Commission (2006). De demografische toekomst van Europa: probleem of uitdaging? [The demographic future of Europe - From challenge to opportunity].

[2] Centraal Bureau voor de Statistiek. Arbeidsdeelname; 15 jaar of ouder [Work participation; 15 years or older]. 2014; Available at: http://statline.cbs.nl/StatWeb/publication/?DM=SLNL&PA=71886ned&D1=18&D2=a&D3=0,11-13&D4=10,74,l&HDR=T,G3&STB=G2,G1&VW=T. Accessed 24 Sept 2014.

[3] Kromer B, Howard D (2013). Labour Force Participation and Work Status of People 65 Years and Older.

[4] Damman M, Henkens K, Kalmijn M (2011). The impact of midlife educational, work, health, and family experiences on men's early retirement. J Gerontol Ser B Psychol Sci Soc Sci.

[5] De Wind A, Geuskens GA, Ybema JF, Blatter BM, Burdorf A, Bongers PM (2014). Health, job characteristics, skills, and social and financial factors in relation to early retirement - Results from a longitudinal study in the Netherlands. Scand J Work Environ Health.

[6] Fischer JAV, Sousa-Poza A (2006). The institutional determinants of early retirement in Europe.

[7] Henkens K, van Dalen H, van Solinge H (2009). De vervagende grens tussen werk en pensioen. Over doorwerken, doorstarten en herintreders [The fading border between employment and retirement. About continuing to work and re-entering employment].

[8] Lund T, Iversen L, Poulsen KB (2001). Work environment factors, health, lifestyle and marital status as predictors of job change and early retirement in physically heavy occupations. Am J Ind Med.

[9] Reeuwijk KG, de Wind A, Westerman MJ, Ybema JF, van der Beek AJ, Geuskens GA (2013). 'All those things together made me retire': qualitative study on early retirement among Dutch employees. BMC Public Health.

[10] Van Rijn RM, Robroek SJ, Brouwer S, Burdorf A (2014). Influence of poor health on exit from paid employment: a systematic review. Occup Environ Med.

[11] Elder GH, Johnson MK, Crosnoe R, Mortimer JT, Shanahan MJ (2003). The emergence and development of life course theory. Handbook of the life course.

[12] Schreurs B, de Cuyper N, van Emmerik IJH, Notelaers G, de Witte H (2011). Job demands and resources and their associations with early retirement intentions through recovery need and work enjoyment. SA J Ind Psychol.

[13] Henkens K, van Solinge H (2013). Returning to Work after Retirement - Who, What and Why?.

[14] Van den Berg TIJ, Elders LAM, Burdorf A (2010). Influence of health and work on early retirement. J Occup Environ Med.

[15] Friis K, Ekholm O, Hundrup YA, Obel EB, Grønbæk M (2007). Influence of health, lifestyle, working conditions, and sociodemography on early retirement among nurses: The Danish nurse cohort study. Scand J Public Health.

[16] Henkens K (1999). Retirement intentions and spousal support: a multi-actor approach. J Gerontol B Psychol Sci Soc Sci.

[17] De Vroom B (2004). The shift from early to late exit: Changing institutional conditions and individual preferences. The case of the Netherlands. In: Maltby T, De Vroom B, Mirabile ML, Overbye E, editors. Ageing and the transtion to retiremen.

[18] Rijksoverheid. Future pension system. Available at: https://www.rijksoverheid.nl/onderwerpen/pensioen/inhoud/toekomst-pensioenstelsel. Accessed 8 June 2016.

[19] De Wind A, Geuskens GA, Reeuwijk KG, Westerman MJ, Ybema JF, Burdorf A (2013). Pathways through which health influences early retirement: a qualitative study. BMC Public Health.

[20] Ybema J, Geuskens G, van den Heuvel S, de Wind A, Leijten F, Joling C (2014). Study on Transitions in Employment, Ability and Motivation (STREAM): The design of a four-year longitudinal cohort study among 15,118 persons aged 45 to 64 years. Br J Med Med Res.

[21] Pearlin LI, Lieberman MA, Menaghan EG, Mullan JT (1981). The stress process. J Health Soc Behav.

[22] Schaufeli WB, Bakker AB, Salanova M (2006). The measurement of work engagement with a short questionnaire: A cross-national study. Educ Psychol Meas.

[23] Ware JE, Kosinski M, Keller SD (1996). A 12-Item Short-Form Health Survey: Construction of Scales and Preliminary Tests of Reliability and Validity. Med Care.

[24] Koppes L, de Vroome E, Mol M, Janssen B, Van den Bossche S (2010). Nationale Enquête Arbeidsomstandigheden 2009: Methodologie en globale resultaten [The Netherlands Working Conditions Survey 2009: Methodology and overall results].

[25] Hildebrandt VH, Bongers PM, van Dijk FJ, Kemper HC, Dul J (2001). Dutch Musculoskeletal Questionnaire: description and basic qualities. Ergonomics.

[26] Karasek R (1985). Job Content Questionnaire and User's Guide.

[27] Karasek R, Brisson C, Kawakami N, Houtman I, Bongers P, Amick B (1998). The Job Content Questionnaire (JCQ): an instrument for internationally comparative assessments of psychosocial job characteristics. J Occup Health Psychol.

[28] Koppes L, de Vroome E, Mol M, Janssen B, Van den Bossche S (2009). Nationale Enquête Arbeidsomstandigheden 2008: Methodologie en globale resultaten [The Netherlands Working Conditions Survey 2008: Methodology and overall results].

[29] Kristensen TS, Hannerz H, Høgh A, Borg V (2005). The Copenhagen Psychosocial Questionnaire - A tool for the assessment and improvement of the psychosocial work environment. Scand J Work Environ Health.

[30] Van Veldhoven M, Dorenbosch L (2008). Age, proactivity and career development. Career Development International.

[31] van den Berg PT (2011). Characteristics of the work environment related to older employees' willingness to continue working: intrinsic motivation as a mediator. Psychol Rep.

[32] McMichael AJ (1976). Standardized mortality ratios and the ‘healthy worker effect’: scratching beneath the surface. J Occup Med.

